# Does the Sole Description of a Tax Authority Affect Tax Evasion? - The Impact of Described Coercive and Legitimate Power

**DOI:** 10.1371/journal.pone.0123355

**Published:** 2015-04-29

**Authors:** Barbara Hartl, Eva Hofmann, Katharina Gangl, Martina Hartner-Tiefenthaler, Erich Kirchler

**Affiliations:** 1 University of Vienna, Vienna, Austria; 2 Vienna University of Technology, Vienna, Austria; Middlesex University London, UNITED KINGDOM

## Abstract

Following the classic economic model of tax evasion, taxpayers base their tax decisions on economic determinants, like fine rate and audit probability. Empirical findings on the relationship between economic key determinants and tax evasion are inconsistent and suggest that taxpayers may rather rely on their beliefs about tax authority’s power. Descriptions of the tax authority’s power may affect taxpayers’ beliefs and as such tax evasion. Experiment 1 investigates the impact of fines and beliefs regarding tax authority’s power on tax evasion. Experiments 2-4 are conducted to examine the effect of varying descriptions about a tax authority’s power on participants’ beliefs and respective tax evasion. It is investigated whether tax evasion is influenced by the description of an authority wielding coercive power (Experiment 2), legitimate power (Experiment 3), and coercive and legitimate power combined (Experiment 4). Further, it is examined whether a contrast of the description of power (low to high power; high to low power) impacts tax evasion (Experiments 2-4). Results show that the amount of fine does not impact tax payments, whereas participants’ beliefs regarding tax authority’s power significantly shape compliance decisions. Descriptions of high coercive power as well as high legitimate power affect beliefs about tax authority’s power and positively impact tax honesty. This effect still holds if both qualities of power are applied simultaneously. The contrast of descriptions has little impact on tax evasion. The current study indicates that descriptions of the tax authority, e.g., in information brochures and media reports, have more influence on beliefs and tax payments than information on fine rates. Methodically, these considerations become particularly important when descriptions or vignettes are used besides objective information.

## Introduction

Official announcements of tax authorities as well as media reports provide taxpayers with descriptions of their tax authority. The question arises how taxpayers respond to such information and whether different descriptions of a tax authority affect taxpayers`beliefs regarding tax authority’s power (coercive power or legitimate power), and most interestingly tax evasion [1]. Taxpayers’ subjective beliefs regarding tax authority’s coercive or legitimate power, may even outweigh economic key determinants [2]. While coercive power bases on frequent audits and severe fines in case of tax evasion, legitimate power rests upon the legitimacy of the position of the authority, its expertise, its dissemination of information, and its ability to be a role model for identification [3].

Field experiments show that audit information letters from the tax authority to taxpayers impact self-reported income [4–6]. Some of these letters can be seen as unintentional manipulation of two forms of power: coercive power and legitimate power. First, taxpayers were informed that both their state and federal tax returns would be closely examined [6]. This description of coercive power such as severity of fines may change taxpayer’s beliefs regarding tax authority`s coercive power and increase tax honesty by leading to an overestimation of the actual audit probability. Second, letters in the field experiment contain department phone numbers to call for information and assistance with tax filing. This assurance of support corresponds with the concept of legitimate power [7]. Strategies of good governance, establishing a customer orientation and focusing on supportive and transparent processes, lead to higher tax compliance [8–10]. These measures of legitimate power promise to be more effective than traditional coercive methods [9,11] because the perception of transparency and fairness induces a feeling of reciprocity at the taxpayers, which in turn enhance tax compliance [12].

The aim of the current study is to analyze whether taxpayers base their decisions to pay taxes honestly on beliefs about tax authority’s power rather than on economic key determinants, and whether the description of a tax authority (as wielding coercive and legitimate power) affects taxpayers’ beliefs and as a result tax evasion, although objective information concerning audit probability and fines is available. Contrary to previous research, the isolated effects of the description of coercive and legitimate power are examined. Further, it is of interest whether these isolated effects of coercive and legitimate power still hold when a tax authority is described as wielding both forms of power simultaneously.

### Theoretical background

The classical economic model of tax evasion [13–15] is the dominant theoretical model in research on tax compliance. In this model, taxpayers are treated as expected utility maximizers, confronted with a decision under risk. Basically, individuals decide whether to evade taxes running the risk of getting audited and fined ending up with less money than if they had declared all income honestly, or to pay honestly, which results in a sure loss. The implementation of audits and fines increases the costs of non-compliance [15]. Thus, audits and fines are researched as authorities’ means for enhancing taxpayers`compliance [6, 16–22]. The probability of an audit is positively related to tax compliance [6,17,21,23–25], as are high fines for tax evasion [17,22].

In the simplest form of the economic model of tax evasion [16], taxpayers receive an amount of income I, and have to decide how much income to report to the tax authority (see also [6]). If income I is reported honestly, the income is taxed according to the tax rate t and taxpayers end up with an income of IH = I—t x I. If taxpayers fully evade taxes, they save money, but run the risk of getting caught, as audits take place with a fixed probability p. When taxpayers are audited, than all unreported income can be discovered and the audit results in a payment of a fine at rate f. The income IC therefore equals IC = I − f x (t x I). When taxpayers have not reported any income to the tax authority and no audit takes place, then they end up with an income of IN = I. Taxpayers are therefore assumed to choose between a sure loss (paying honestly, E(IH) = I—t x I) or the probability of a gain or a major loss according to the expected utility function of E(I) = p(IC) + (1-p) (IN).

Literature reviews report inconsistent empirical findings concerning the relationship between audits, fines, and tax evasion [18,26,27]. Although laboratory experiments have consistently supported the positive impact of audits on tax behavior, levels of tax honesty in field experiments are far higher than a simple risk-return model would predict [28]. In almost all countries in the world the audit probability is low, which should result in low levels of tax compliance. Contrary to this economic assumption, tax honesty is still high in most countries [29].

These findings may be due to the fact that individuals find it hard to deal with uncertainty. Several studies show that taxpayers poorly predict audit probabilities and fine rates [18,30,31]. Rather than on objective key economic figures, taxpayers seem to base their tax behavior on their subjective beliefs regarding tax authority’s ability to detect evasion, that is the tax authority’s power [2,32]. Prior research has shown different ways by which taxpayers become aware of enforcement strategies of the tax authority [33]. According to these insights, taxpayers pay attention to official information disseminated by the tax authority as well as unofficial information. It is therefore of relevance to investigate how the description of the tax authority modifies taxpayers’ beliefs of tax authority’s power and tax evasion.

A tax authority can be believed to be powerful, holding coercive power or legitimate power. Descriptions of coercive power comprise the severity of fines for tax evasion [10,34]. In such a case taxpayers overestimate the probability of audits and fine rates, even when fully informed, so that there is far less tax evasion than predicted by the economic model [16]. Beliefs on tax authorities’ coercive power instead of accurate information on audits and fines rates should act as an indicator for the estimation of audit probability and fines and therefore affect tax compliance [35]. Describing a tax authority as wielding high coercive power should positively impact tax honesty.

The perception of legitimate power origins from taxpayers’ conviction that the tax authority holds expertise [10]. Savvy information for taxpayers to pay their taxes correctly prompts a perception of legitimate power. Another source of legitimate power roots in the feeling of identification with the tax authority and the authority’s goals. Legitimate power also stems from the perception that the tax authority is in a (legal) position that gives authority over taxpayers. Beliefs on tax authorities’ legitimate power should increase tax honesty as they lead to perceptions of fairness and transparency, and in turn call for reciprocal behavior (“You scratch my back, and I’ll scratch yours”, [36]). Taxpayers are more likely to report their income honestly when they think that the tax authority is doing a good job [1]. When the tax authority works in a way that is beneficial for the taxpayers (e.g., provides services), they cooperate in paying taxes even when defection would rationally be the best option in the short-term [12,26]. The impact of perceived legitimate power on tax compliance has only been investigated indirectly, for instance, by investigating the impact of service considerations on tax payments [7] or by investigating the effect of legitimate power on intended tax compliance [10]. Empirical evidence shows that legitimate power enhances tax payments. However, it is relevant to examine if descriptions of legitimate tax authorities initiate beliefs on legitimate power and affect tax evasion even in cases where objective information about economic key figures are present.

It is necessary to examine the isolated effects of descriptions of coercive and legitimate power, as they are supposed to impact tax evasion in different ways. Up to now, field experiments using announcements and tax letters do not explicitly address coercive or legitimate power or only trigger the perception of both forms of power [6]. Likewise, descriptions or vignettes on power of the tax authority used in experiments with behavioral data do not explicitly differentiate between coercive and legitimate power [2,37].

Different qualities of power do not exist independently of each other [38]. Tax authorities usually do not choose one set of policies over another, but rather set measures of both, coercive and legitimate power [10]. A few studies address interaction effects of different types of social power in an organizational context [39,40]. It was shown that employees comply with managerial directions in particular when their supervisors exert coercive as well as legitimate power [41]. In the context of taxes, the perception of a high audit probability and severe fines (coercive power) may be of little deterrent value when taxpayers think that an audit will fail to uncover tax evasion [18]. Although the combination of coercive power and legitimate power might lead to a reduction of beliefs of coercive measures, additional legitimate power is discussed to signal efficiently applied audits to discover non-compliance, as experts are at work. Research has shown, that if sanction mechanisms are believed to be fair (that is legitimate), cooperation increases [42]. Hofmann et al. [10] assume that in the tax context coercive and legitimate power exercised in combination generate the highest degree of intended tax honesty because the tax authority is perceived as a legitimate and expert power holding authority. In this vein, the joint description of coercive and legitimate power may have a higher impact on tax payments than if only one quality of power is perceived.

Although descriptions of coercive and legitimate power may outweigh objective information, the effect could differ according to taxpayers’ prior experience with the tax authority. External factors, like a change of government after an election or a revolution, can prompt a change of the belief of the intensity of power. How do taxpayers react to such a change of power? As tax authorities worldwide start changing their policy to enhance compliance by using supportive procedures rather than means of enforcement, it is relevant to further research how taxpayers adjust their beliefs and react to such a contrast of power. As descriptions on prior events are used to qualify current judgments, a change could foster contrast effects [43,44]. A change of the description of the tax authority will lead to a comparison process, in which new evidence on the tax authority will be evaluated in contrast to the previous description. For instance, after experiencing a contrast from high to low coercive power of the tax authority, taxpayers may react with less compliance than they would without the specific prior descriptions of power, even if economic key figures remain constant. On the other hand, a contrast from high to low legitimate power indicates that the tax authority has reduced its services, which leads to decreased reciprocal behavior. Taxpayers experiencing these contrasting descriptions might be less compliant than taxpayers who do not undergo this change but constantly face descriptions of low legitimate power. The contrast of described power (low to high power; high to low power) may therefore impact tax evasion.

### The Current Studies

Based on the inconsistent findings regarding the relationship between the severity of fines and tax evasion, we first conduct an experiment, in which we examine the impact of information on one economic key determinant, i.e., fine rates (0.5 vs. 1 vs. 2 times the evaded amount) on tax evasion, the impact of beliefs regarding tax authority’s power on tax evasion and whether beliefs are based on the information about the fine rate (Experiment 1). Further, we extend previous research by modifying participants’ beliefs regarding tax authority’s power with varying description about the tax authority, and investigate whether the descriptions of the authority affect tax evasion. Three experiments are conducted with varying descriptions of the tax authority (Experiment 2–4). The procedures were similar and economic key figures, such as audit probability and fine rate, were kept constant throughout the three experiments. In particular, we investigate whether the description of a tax authority exerting low versus high coercive power (Experiment 2) or low versus high legitimate power (Experiment 3) impacts tax evasion while economic key figures are held constant. Building on these findings, we examine whether the combination of high coercive and legitimate power induce higher tax payments than if the intensity of only one quality of power is described as high (Experiment 4). A possible contrast effect of presentations of the tax authority is examined in the experiments 2, 3, and 4.

### Ethic Statement

All four experiments reported here were conducted in respect to the Declaration of Helsinki (revised 1983) and local guidelines of the Faculty of Psychology, University of Vienna. According to the Austrian Universities Act 2002 (UG2002), only medical universities are required to appoint ethics committees for medical research. Therefore, no ethical approval was required for the present study. The majority of participants were recruited via an online data bank of the Department of Applied Psychology, for which they had to provide their written consent to participate in studies. All participants were invited to the laboratory of the Faculty and gave their verbal consent to participate in the study. Information about the duration, the tasks, the payment, and the confidentiality was provided to participants prior to attending the experiments. All participants voluntarily took part in the experiment and could withdraw at any time during the experiment with no further consequences. Data were collected anonymously and no harming procedures were used. The experiments are part of a project proposal approved by three international scientific peer-reviewers from the field for the Austrian Science Fund (FWF).

## Experiment 1: Fine Rate

### Materials and Methods

#### Participants

Overall, 108 students (32% males, *M*[age] = 23.96, *SD* = 5.92) majoring in different fields from management to biology (not acquainted with the tested theories and hypotheses) took part in the study. Thirty-eight percent of participants had no experience with the tax authority; the others reported at least some contact.

#### Procedure

The experiment was conducted with the software z-Tree [45]. Participants were invited to the laboratory and randomly assigned to one of three conditions, manipulating the fine rate in case of detected tax evasion (0.5 vs. 1 vs. 2 times the evaded amount).

#### Introduction

At the beginning of the experiment, participants were introduced to the rules of the experiment and informed about audit probability (kept constant in all conditions) and fine rate (0.5 vs. 1 vs. 2 times the evaded amount). A numeric example helped to understand the information.

In the following you are taking part in an experiment on tax behavior which takes 20 periods. In each period a certain income is allocated to you, of which you have to pay taxes. The tax rate is 40% of your income. In each period your final income is the result of the allocated income minus the taxes paid. At the end of the experiment one period will be selected randomly. The income that you have gained in this period will be paid to you by the experimenter. Additionally, for each period there exists a tax audit probability of 15%. In case you are audited and you have evaded taxes, you have to pay back the evaded amount plus a fine of [0.5] [1] [2] time/s the evaded amount.

After the introduction, participants were asked to imagine being a self-employed taxpayer in a fictitious state who has to pay taxes. They also received the information that only at the end of the experiment they get to know if and when an audit had taken place. After every fifth period, participants were briefly asked to remember that they are self-employed and that they will have to pay taxes in Chomland for the next remaining years (periods). We repeated the following analysis, controlling for a possible effect of the reminder. The analysis showed that the reminder has no effect on the tax payments, revealing the same results.

#### Experimental task

In each of the 20 periods, participants received varying incomes in experimental currencies (ECU) on which they had to pay taxes. Every participant received the same amount of money in every round, ranging from 50,000 ECU to 95,000 ECU (sequence was set before the experiment). They were asked to indicate how much tax they will pay from the allocated income. For further analyses, the relative tax compliance (ranging from 0 to 1) was used.

#### Questionnaire

After the 20^th^ period, participants filled in a questionnaire to assess their beliefs about tax authorities’ coercive (4 items) and legitimate power (22 items). Reliability of scales, tested with Cronbach α, was .85 for coercive power and .90 for legitimate power. The scale legitimate power compounds four subscales (legitimacy, expertise, information, identification). For the sake of simplicity and due to the measurement model the subscales were combined to one scale. All items can be found online in supplementary material (see S1 Table).

#### Remuneration

At the end of the experiment, participants were remunerated for participation. One period out of 20 was randomly selected and participants were remunerated according to their decisions in this period. The participants’ earnings depended on their tax payments, random tax audits and the respective fines in case of detected tax evasion (participants received on average 2.53 EUR or 3.16 USD, respectively).

### Results

#### Fine rate on tax evasion

To test whether different fine rates have an impact on tax evasion, the three conditions (0.5 vs. 1 vs. 2 times the evaded amount) are included as dummy variables in a repeated measures regression, analyzing all 20 periods of taxpaying (*F*(17, 106) = 2.18, *p* = .008, *R* ^2^ = .18). Fine rate 1 serves as reference group. The analysis reveals no significant differences between 1 time and 0.5 times the evaded amount, β = -.04, *t*(106) = -0.55, *p* = .58 and 1 time and 2 times the evaded amount, β = .02, *t*(106) = 0.32, *p* = .75. Regardless of the manipulation, tax evasion is equally low when the fine was 0.5 times (*M*_*0*.*5x*_ = .82, *SD* = .29), 1 time (*M*_*1x*_ = .84, *SD* = .23) or 2 times the evaded amount (*M*_*2x*_ = .85, *SD* = .23). The amount of fine (0.5 vs. 1 vs. 2 times fine) has no impact on tax evasion.

#### Beliefs on tax evasion

To test whether beliefs about tax authority’s coercive and legitimate power impacts tax evasion, a repeated measure regression is conducted, *F*(18, 105) = 4.14, *p* < .001, *R* ^2^ = .31. The analysis reveals a significant main effect of coercive power, β = .83, *t*(105) = 2.19, *p* = .03 and significant main effect of legitimate power, β = .65, *t*(105) = 2.37, *p* = .02, but no significant interaction effect of coercive and legitimate power (*p* = .10). As expected, the perception of the tax authority as wielding high coercive or high legitimate power leads to higher tax payments.

#### Fine rate on beliefs

A MANOVA reveals no significant effect of different fine rates on participants`beliefs regarding tax authority’s coercive power (*F*(2, 104) = 0.77, *p* = .47) or legitimate power (*F*(2, 104) = 0.47, *p* = .63). Regardless of severity of fines, the beliefs of coercive power (*M*_*0*.*5x*_ = 4.50, *SD* = 1.46; *M*_*1x*_ = 4.49, *SD* = 1.33; *M*_*2x*_ = 4.86, *SD* = 1.57) and legitimate power (*M*_*0*.*5x*_ = 4.40, *SD* = 0.84; *M*_*1x*_ = 4.60, *SD* = 0.93; *M*_*2x*_ = 4.58, *SD* = 1.03) are equally high in all three conditions. This indicates that tax evasion is related to participants`beliefs regarding tax authority’s power, but the manipulation of fine rates does not affect the beliefs of coercive and legitimate power.

## General Method for Experiment 2, 3, and 4

In experiment 2, 3, and 4, we examine whether descriptions of the tax authority affect participant’s beliefs regarding tax authority’s power and further affect tax evasion, while all economic key figures, including fine rate are held constant.

### Procedure

The following procedure was identical for experiment 2, 3, and 4. The experiments were conducted with the software z-Tree [45] and took place in the laboratory.

#### Economic determinants

Equal to experiment 1, participants were introduced to the rules and informed about audit probability and fine rate at the beginning of the experiments. A numeric example helped to internalize the information.

In the following you are taking part in an experiment on tax behavior which takes 40 periods. In each period a certain income is allocated to you, of which you have to pay taxes. The tax rate is 40% of your income. In each period your final income is the result of the allocated income minus the taxes paid. At the end of the experiment one period will be selected randomly. The income that you have gained in this period will be paid to you by the experimenter. Additionally, for each period there exists a tax audit probability of 15%. In case you are audited and you have evaded taxes, you have to pay back the evaded amount plus a fine of 1 time the evaded amount.

#### Introduction to the fictitious scenario

Participants were asked to imagine being a self-employed taxpayer in a fictitious state who has to pay taxes. They also received the information that only at the end of the experiment they get to know if and when an audit had taken place.

Imagine that you are a citizen in the fictitious state Chomland. You are self-employed and you will have to pay taxes in Chomland for the next 40 years (40 periods). In Chomland, you only get to know if and when a tax audit took place after employment has ended (40 periods).

#### Manipulation I: Description of tax authority

The description of the tax authority in the fictitious state differed between the three experiments. After the introduction to the fictitious state, participants read a description of the tax authority, which was holding different qualities of power. The description contained data about the way in which the authority makes sure that taxpayers comply. Coercive power and legitimate power were manipulated either separately (Experiment 2 and 3) or in combination (Experiment 4). After every fifth period, participants were reminded of the features of the respective tax authority by means of sentences repeated from the description of the tax authority (i.e. short sequences repeated from the description they had received in the beginning; additional analysis for all three experiments showed that the reminder had no effect on the tax payments). To check whether the description of the tax authority affects participants’ beliefs about tax authorities power, participants filled in a questionnaire assessing their perception of wielded coercive and legitimate power by the tax authority twice, after the 20^th^ period (first sequence) and the 40^th^ period (second sequence) of the experimental task (Table 1). Reliability of scales was tested with Cronbach α, ranging from .89 to .97 over the experiments.

**Table 1:** Manipulation of coercive and legitimate power before (Periods 1–20) and after the change in the tax authority (Periods 21–40).

	Experimental conditions		Control conditions	
	Periods in experiment		Periods in experiment	
	1–20	21–40	1–20	21–40
**Experiment 2**	low coercive	high coercive	high coercive	high coercive
	high coercive	low coercive	low coercive	low coercive
**Experiment 3**	low legitimate	high legitimate	high legitimate	high legitimate
	high legitimate	low legitimate	low legitimate	low legitimate
**Experiment 4**	high coercive/ low legitimate	high coercive/ high legitimate	high coercive/ high legitimate	high coercive/ high legitimate
	low coercive/ high legitimate	high coercive/ high legitimate		
	high coercive/ high legitimate	high coercive/ low legitimate	high coercive/ low legitimate	high coercive/ low legitimate
	low coercive/ low legitimate	high coercive/ low legitimate		
	high coercive/ high legitimate	low coercive/ high legitimate	low coercive/ high legitimate	low coercive/ high legitimate
	low coercive/ low legitimate	low coercive/ high legitimate		
	high coercive/ low legitimate	low coercive/ low legitimate	low coercive/ low legitimate	low coercive/ low legitimate
	low coercive/ high legitimate	low coercive/ low legitimate		

#### Manipulation II: Contrast of power

In all three experiments, control conditions and experimental conditions were realized. After the 20^th^ period (first sequence), participants were informed that due to a change of the government, the intensity of power of the tax authority had changed. Participants in the experimental conditions received a different description of this new tax authority contrasting the first description. In the control condition, the hypothetical tax authority was the same in the first and second sequence. Table 1 provides an overview of all experimental and control conditions for the three experiments.

#### Experimental task

Equal to experiment 1, in each period, participants received varying incomes in experimental currencies (ECU) on which they had to pay taxes. They were asked to indicate how much taxes they will pay. For further analyses, the relative tax compliance (ranging from 0 to 1) was used.

#### Remuneration

One period out of 40 was randomly selected and participants were remunerated according to their decisions in this period. The participants’ earnings depended on their tax payments, random tax audits and the respective fines in case of detected tax evasion (participants received on average 6.20 EUR or 8.18 USD, respectively).

## Experiment 2: Coercive Power

### Materials and Methods

#### Participants

Overall, 120 students (64% males, *M*[age] = 24.48, *SD* = 5.85) majoring in several different fields from management to biology (not acquainted with the tested theories and hypotheses) took part in the study. Forty-seven percent of participants had no kind of experience with the tax authority; the others reported at least some contact with them.

#### Procedure

Participants were randomly assigned to one of four conditions (two experimental conditions: low coercive power (lcp) → high coercive power (hcp), hcp → lcp; two control conditions: lcp → lcp, hcp → hcp), in which the tax authority were described as holding low and/or high coercive power.

#### Low/high coercive power manipulation

The tax authority of Chomland calculated that the tax revenue was about 200 billion ECU for the past year. Of this income, about 0.09/2 billion ECU was spent on tax audits and punishments of taxpayers. In general, the tax authority is well known for its mild/severe punishments. Since it has so far rarely/always conducted strict audits, the taxpayers feel little/very forced to cooperate.

The tax authority consists of employees who work with lax/strict audits.

For the tax authority, the severity of punishment for tax evasion is of low/high importance. It works on the basis of lax/strict control measures. Its working principles are based little/particularly on the penalties for tax evasion.

After every fifth period, participants received a reminder of the features of the respective tax authority by means of sentences repeated from the description of the tax authority. For instance, participants in the condition, in which the tax authority held high coercive power received the following reminder:

Please remember! You are a citizen in Chomland, in which the tax authority is well known for its severe punishment. Tax auditors work on the basis of lax/strict control measures.

Additionally, during their ‘experimental’ life as a taxpayer, power of the tax authority changed in the experimental conditions from low/high coercive power to high/low coercive power (Table 1).

### Results

#### Coercive power on beliefs

A repeated measures regression (*F*(27, 119) = 31.70, *p* < .001, *R* ^2^ = .66) reveals a significant main effect of intensity of power, β = .78, *t*(119) = 15.41, *p* < .001 and a significant interaction effect of intensity of power and sequence, β = .10, *t*(119) = 3.24, *p* = .002. As expected, the description of the tax authority as wielding low or high coercive power affects the participant’s beliefs regarding the tax authority’s power. Whereas the belief of low coercive power is equally low in the first sequence (*M* = 2.60, *SD* = 1.34) and the second sequence (*M* = 2.52, *SD* = 1.67), high legitimate power is perceived to be lower in the first sequence (*M* = 5.51, *SD* = 1.37) than in the second sequence (*M* = 6.06, *SD* = 0.87). Thus, descriptions of low and high coercive power initiate respective beliefs.

#### Coercive power on tax evasion

**Fig 1:**
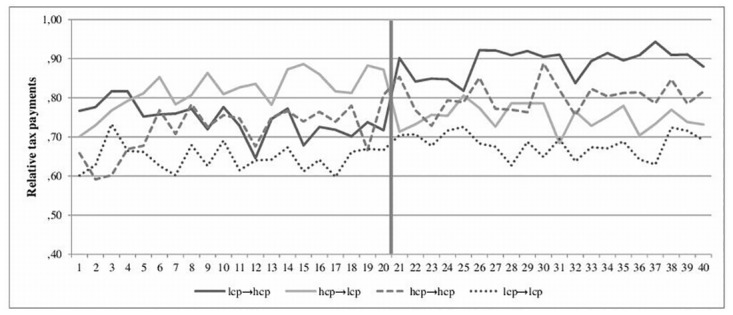
Experiment 2. The impact of low and high coercive power on relative tax payments before and after the change in tax authority. Note. lcp … low coercive power, hcp … high coercive power.

To test whether coercive power has an impact on tax payments, again a repeated measure regression was conducted, analyzing all 40 periods of taxpaying. The model with all variables significantly predicts tax payments, *F*(26, 119) = 5.10, *p* < .001, *R* ^2^ = .15. As expected, there is a significant main effect of intensity of power, β = .14, *t*(119) = 2.64, *p* = .009, with higher tax payments in conditions with high coercive power than with low coercive power. In addition, there is a significant main effect of sequence, β = .07, *t*(119) = 3.22, *p* = .002, with higher tax payments in the second sequence than in the first sequence (Fig 1). The other main effects and interaction effects do not reach significance, *p* > .20. Therefore, descriptions of coercive power impact tax payments. Table 2 provides the results for the repeated measure regression for all three experiments.

**Table 2:** Repeated measure regression predicting relative tax payments in Experiment 2, 3, and 4.

	Experiment					
	Experiment 2		Experiment 3		Experiment 4	
Predictor	Δ*R* ^2^	β	Δ*R* ^2^	β	Δ*R* ^2^	β
Model	.15***		.22***		.12***	
Coercive Power (CP)		.14**				.20***
Legitimate Power (LP)				.33***		.12***
Sequence		.07**		.02		.04***
Condition		.10		.05		-.04
CP X LP						.01
CP X Sequence		.04				.04
CP X Condition		.02				.02
LP X Sequence				.03		.01
LP X Condition				-.05		.02
Sequence X Condition		-.01		-.03		.02
CP X LP X Sequence						-.01
CP X LP X Condition						-.09**
CP X Sequence X Condition		.00				.03
LP X Sequence X Condition				-.02		-.01

Note. Repeated measure regression was clustered at the individual level, controlling for gender, age, income, nationality, employment, conditions of work and experience with tax authority.

**p < .01,

***p < .001

#### Contrast effect of coercive power

To test for a contrast effect of descriptions, all conditions are compared with an ANOVA due to the tax payments in the second sequence, i.e., the last 20 periods. The analysis shows that overall, the four treatment conditions significantly differed (lcp → hcp, hcp → lcp, lcp → lcp, hcp → hcp), *F*(3, 116) = 3.52, *p* = .02, η_p_^2^ = .08. But post hoc tests show that the comparison of the relevant experimental and control conditions in the second sequence (lcp → hcp versus hcp → hcp, hcp → lcp versus lcp → lcp) reveals no significant result: Participants who experienced a contrast from low to high coercive power declare the same amount of income as participants in the control conditions, who experienced only high coercive power (*p* = .18). Equally, a change from high to low coercive power does not lead to lower tax payments than when the tax authority wielded only low coercive power (*p* = .30). Therefore, a change in coercive power does not lead to a contrast effect in beliefs.

## Experiment 3: Legitimate Power

### Materials and Procedures

#### Participants

Students (*N* = 130, 60% males, *M*[age] = 24.40, *SD* = 4.89) majoring in different fields took part in the study. Most participants had at least some contact with the tax authority, but 43% of participants reported to have no experience with them.

#### Procedure

For Experiment 3, basically the same procedure as in Experiment 2 was applied. However, the description of the tax authorities depicted legitimate power instead of coercive power as in Experiment 2. Again, participants were randomly assigned to one of four conditions (two experimental conditions: low legitimate power (llp) → high legitimate power (hlp), hlp → llp; two control conditions: llp → llp; hlp → hlp), in which the fictitious tax authority held either low or high legitimate power.

#### Low/high legitimate power manipulation

The tax authority of Chomland calculated that the tax revenue was about 200 billion ECU for the past year. Of this income, about 0.09/2 billion ECU was spent on the training of employees of the tax authority and on the advising of taxpayers.

In general, the tax authority is little/very appreciated for its work. As it has provided bad/good service so far, the taxpayers feel little/much obliged to cooperate.

The tax authority consists of early school leavers/experts who work with non-professional/professional advice. For the tax authority, the accuracy of the tax returns is of low/high importance. It works on the basis of unlawful/lawful measures. Its working principles are based little/particularly on the traceability of decisions.

Similar to Experiment 2, the power of the tax authority changed in the experimental conditions during the life as taxpayers from low/high legitimate power to high/low legitimate power (Table 1).

### Results

#### Legitimate power on beliefs

A repeated measures regression (*F*(27, 129) = 22.17, *p* < .001, *R* ^2^ = .59) reveals a significant main effect of intensity of power, β = .69, *t*(129) = 13.23, *p* < .001 and a significant interaction effect of intensity of power and sequence, β = .14, *t*(129) = 3.85, *p* < .001. As expected, the description of the tax authority as wielding low or high legitimate power affects the participants’ beliefs regarding the tax authority’s power. Whereas low legitimate power is perceived as significantly lower in the first sequence (*M* = 3.48, *SD* = 1.64) than in the second sequence (*M* = 3.84, *SD* = 1.72), high legitimate power is perceived as equally high in the first sequence (*M* = 4.51, *SD* = 1.57) and in the second sequence (*M* = 4.76, *SD* = 1.39). Thus, descriptions of low and high legitimate power initiate respective beliefs.

#### Legitimate power on tax evasion

**Fig 2:**
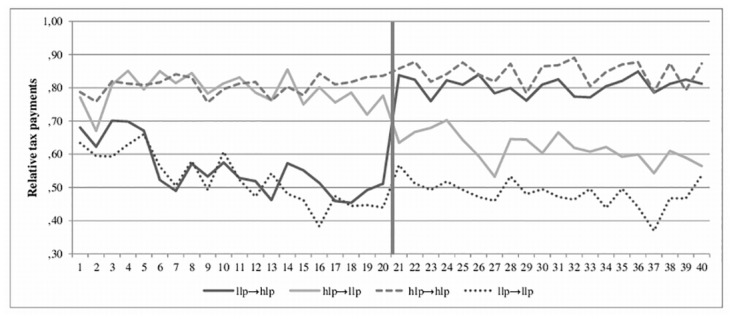
Experiment 3. The impact of low and high legitimate power on relative tax payments before and after the change in tax authority. Note. llp … low legitimate power, hlp … high legitimate power.

Again, to test whether legitimate power has an impact on tax payments, a repeated measures regression is conducted, analyzing all 40 periods of taxpaying (Table 2). The model with all variables significantly predicts tax payments, *F*(27, 129) = 9.45, *p* < .001, *R* ^2^ = .22. As expected, there is a significant main effect of intensity of power, β = .33 *t*(129) = 6.86, *p* < .001, with higher tax payments in conditions with high legitimate power than low legitimate power (Fig 2). The other main effects and interaction effects do not reach significance, *p* > .22. Therefore, descriptions of legitimate power increase tax payments.

#### Contrast effect of legitimate power

Again, the impact of the contrast of power intensity on tax payments is tested with an ANOVA to examine the difference between the tax payments in all four treatment conditions (llp → hlp, hlp → llp, llp → llp, hlp → hlp) in the second sequence. The analysis shows that overall, the treatment conditions significantly differ, *F*(3, 126) = 11.32, *p* < .001, η_p_^2^ = .21. Again, the post hoc analysis comparing the relevant experimental and control conditions (llp → hlp versus hlp → hlp, hlp → llp versus llp → llp) reveals no significant result: Participants in the experimental condition, who experienced a change from low to high legitimate power, do not pay significantly more taxes than participants in the control condition who experienced only high legitimate power (*p* = .59). Contrary to expectation, results revealed a tendency that a change from high to low legitimate power leads to higher tax payments (*M* = .62, *SD* = .29) than if the tax authority wields only low legitimate power (*M* = .48, *SD* = .35; *p* = .06).

## Experiment 4: Coercive and Legitimate Power Manipulated Combined

Experiment 2 and 3 confirmed that descriptions of coercive power or legitimate power have an impact on tax payments: in Experiment 4, both forms of power were combined with the same design and material as in Experiments 2 and 3.

### Materials and Procedures

#### Participants

Overall, 368 students (34% males, *M*[age] = 24.26, *SD* = 5.56) majoring in different fields participated in the experiment. Again, 41% of participants had no experience with the tax authority, the others mainly very little experience.

#### Procedure

For Experiment 4, basically the same procedure as in the other experiments was applied. The description of the tax authority differed only in the combined display of coercive and legitimate power of the tax authority. Participants were randomly assigned to one of twelve treatment conditions (eight experimental conditions; four control conditions), in which the fictitious tax authority are described as holding low or high coercive power and low or high legitimate power.

#### Low/high coercive power and *low/high legitimate power* manipulation

The tax authority of Chomland calculated that the tax revenue was about 200 billion ECU for the past year.

Of this income, about 0.09/2 billion ECU was spent on tax audits and punishments from taxpayers and about *0*.*09/2* billion for the training of employees of the tax authority and on the advising of taxpayers.

In general, the tax authority is known for its low/high penalties for tax evasion, and is *little/very* appreciated for its work. It works on the basis of *unlawful/lawful* measures as well as lax/strict control measures. Since so far, it has rarely/always conducted strict audits, the taxpayers feel little/very forced to cooperate.

The tax authority consists of employees who work with lax/strict controls.

In addition, the tax authority consists of *early school leavers/experts* who work with *non-professional/professional* advice. Its working principles are based *little/particularly* on the traceability of its decisions and little/particularly on the penalties for tax evasion.

As the authority has provided *bad/good* service so far, the taxpayers feel *little/much* obliged to cooperate.

Overall, for the tax authority the severity of punishments for tax evasion is of little/high importance and the accuracy of the tax returns is of *little/high* importance

In the experimental conditions, the tax authority was described as changing after 20 periods from, e.g., high coercive power and low legitimate power to high coercive power and high legitimate power (Table 1).

### Results

#### Coercive and legitimate power on beliefs

A repeated measure regression (*F*(34, 367) = 40.74, *p* < .001, *R* ^*2*^ = .63) reveals a significant main effect of intensity of coercive power, β = .77, *t*(367) = 25.44, *p* < .001 and a significant main effect of sequence, β = .07, *t*(367) = 3.77, *p* < .001. As expected, the description of the tax authority as wielding low or high coercive power affects the participant’s beliefs regarding the tax authority’s power. The perception of coercive power was lower in the first sequence (*M* = 4.06, *SD* = 1.90) than in the second sequence (*M* = 4.35, *SD* = 2.06).

The analysis for legitimate power (*F*(34, 367) = 19.80, *p* < .001, *R* ^2^ = .50) reveals a significant main effect of intensity of legitimate power, β = .62, *t*(367) = 16.87, *p* < .001 and a significant interaction effect of intensity of legitimate power and condition, β = .13, *t*(367) = 3.41, *p* = .001. In the experimental conditions, low legitimate power is perceived as significantly lower (*M* = 3.11, *SD* = 0.94) than in the control conditions (*M* = 3.38, *SD* = 1.05), whereas high legitimate power is perceived as higher (*M* = 5.05, *SD* = 1.04) in the experimental conditions than in the control conditions (*M* = 4.79, *SD* = 0.87). Thus, descriptions of coercive and legitimate power induce respective beliefs.

#### Coercive and legitimate power on tax evasion

**Fig 3:**
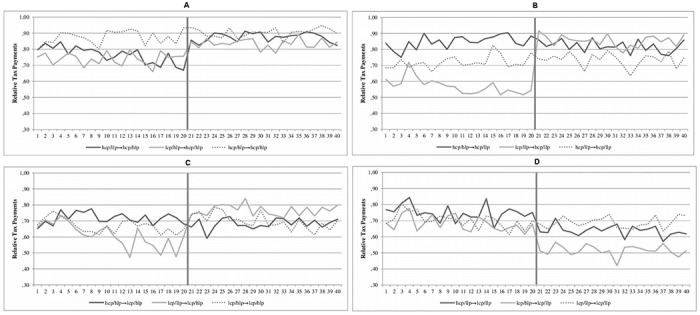
Experiment 4. The impact of coercive power and legitimate power on relative tax payments. (a) Relative tax payments in the experimental conditions and control conditions with high coercive power and high legitimate power in the second sequence. (b) Relative tax payments in the experimental conditions and control conditions with high coercive power and low legitimate power in the second sequence. (c) Relative tax payments in the experimental conditions and control conditions with low coercive power and high legitimate power in the second sequence. (d) Relative tax payments in the experimental conditions and control conditions with low coercive power and low legitimate power in the second sequence. Note. lcp … low coercive power, hcp … high coercive power, llp … low legitimate power, hlp … high legitimate power

To test the impact of the description on tax payments a repeated measures regression is conducted, analyzing all 40 periods of taxpaying (Table 2). The model with all variables significantly predicts tax payments, *F*(34, 367) = 4.86, *p* < .001, *R* ^2^ = .12. As expected, there is a significant main effect of coercive power, β = .20 *t*(367) = 5.57, *p* < .001, as well as legitimate power, β = .12 *t*(367) = 3.04, *p* = .003. The description of high coercive power leads to significant higher tax compliance (*M* = .81, *SD* = .32) than the description of low coercive power (*M* = .66, *SD* = .37). Likewise, a tax authority wielding high legitimate power leads to significant higher tax compliance (*M* = .77, *SD* = .34) than a tax authority wielding low legitimate power (*M* = .70, *SD* = .34). Additionally, a significant three-way interaction effect of intensity of coercive power, legitimate power and condition, β = -.09, *t*(367) = -2.88, *p* = .004 is found. In the control conditions, the combination of high coercive and high legitimate power leads to significantly higher tax payments (*M* = .89, *SD* = .23) than when only one quality of power was high (*M*_*hcp*_ = .73, *SD*_*hcp*_ = .35; *M*_*hlp*_ = .68, *SD*_*hlp*_ = .37). In contrast, in the experimental conditions, the combination of low coercive and low legitimate power leads to significantly lower tax payments (*M* = .58, *SD* = .38) than when one quality of power is applied (*M*_*hcp*_ = .80, *SD*_*hcp*_ = .31; *M*_*hlp*_ = .72, *SD*_*hlp*_ = .36). The other main and interaction effects do not reach significance, *p* > .22. Therefore, simultaneous descriptions of coercive and legitimate power lead to an increase of tax payments. Fig 3a–3d provide an overview of the relative tax payments in all 40 rounds, grouping together the experimental conditions with the corresponding control conditions.

#### Contrast effects of coercive and legitimate power

The impact of a contrast of power is tested with an ANOVA comparing tax payment in the second sequence. The analysis shows that overall, the treatment conditions significantly differ, *F*(11, 356) = 4.98, *p* < .001, η_p_^2^ = .13. A post hoc test shows that participants who experienced a change from ‘low coercive/high legitimate power’ to ‘low coercive/low legitimate power’ declare less income (*M* = .52, *SD* = .37) than the control group ‘low coercive/low legitimate power’ (*M* = .69, *SD* = .27; *p* = .02). All other relevant comparisons show no significant results, *p* > .24. Thus, from all eight possible contrasting descriptions only one has an impact, i.e., the contrast from high to low legitimate power when it is combined with low coercive power.

## General Discussion

Based on the neo-classical economic model of tax evasion [13–15], the severity of fines is one key determinant for taxpayers’ decisions to evade taxes. When integrating the economic determinants of Experiment 1 (40% tax rate; 15% audit probability; 0.5 vs. 1 vs. 2 times fine) into the economic model of tax evasion [16], the rational decision should always be to fully evade taxes, as the expected utility of paying honestly is always lower. Nevertheless, tax evasion is low in all conditions, regardless of the amount of fines. It seems that participants base their tax decision on beliefs regarding tax authority’s power rather than on objective information about the fine rate. This finding is in line with earlier research showing that taxpayers find it hard to deal with economic key figures [18,30,31]. Additional experiments were conducted to examine whether descriptions of the tax authority affect participants’ beliefs regarding tax authority’s power and further affect tax evasion, while all economic key figures, including the fine rate are held constant. Three laboratory experiments confirmed that the description of a tax authority and therefore additional information about coercive and legitimate power has an effect on beliefs on tax authority’s power and most interestingly on tax payments. The positive impact of coercive power is in line with earlier research that stresses the effectiveness of informing taxpayers about coercive means [4–6]. Further, this result extends previous research by showing that even when taxpayers have objective knowledge of audit probability and severity of fines, additional coercive information has an impact on tax evasion. Current findings therefore underline the assumption that subjective beliefs on the probability may be more important for understanding tax evasion than objective criteria [2,18,30]. The impact of the description of legitimate power on tax compliance supports the assumption that the perception of service orientation leads to reciprocal behavior [1,12]. Thus, taxpayers are more likely to report their income honest, when they think that the tax authority works in a way that is beneficial for them. Current findings show that this effect still holds despite available information about economic key figures.

**Fig 4:**
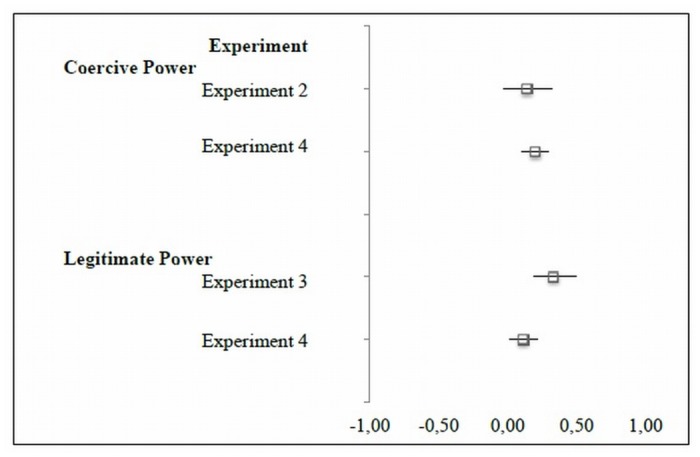
Forest Plot of regression coefficients for coercive power and legitimate power.

An additional objective of the current paper was to investigate possible interaction effects of tax authority’s power. A very important and new finding is that the effect of coercive and legitimate power on tax payments still holds when both qualities of power are applied. However, unlike assumed by previous studies on intended tax compliance [10], legitimate power did not alter the effect of coercive power. Both qualities of power have a similar strong and independent impact on tax compliance (Fig 4).

The current study analyzes possible contrast effects of the description of a tax authority. Contrary to expectations, the contrast of descriptions has little impact on tax evasion. A contrast effect was not found when coercive power and legitimate power are described solely. The description of both qualities of power only leads to a contrast effect in one of eight conditions. When the tax authority is described as holding low coercive power, a decrease in legitimate power leads to higher tax evasion than in the control condition. Future research needs to clarify if and why a decline in perceived legitimacy is more severe for tax compliance than a decline in perceived coercion. For that, additional laboratory experiments and field experiments are needed to clarify this important practical impact.

Like all laboratory experiments, the current study has some limitations; the ecological validity of the results can be questioned. The fact that the amount of tax payments were unrelated to the well-being of the ‘society’ of the fictitious country does not fully represent the situation taxpayers experience in the field. Nevertheless, considering this fact, the results might become even more meaningful. In the current study, evading taxes was not harming anyone and would therefore certainly be the profit-maximizing strategy. However, in no experimental condition did average tax contributions ever drop below 40%; actually, they even reached 94% in one treatment condition. Concerning the interpretation of the results, it has to be taken into account that additional information on tax authority’s coercive power may have increased uncertainty, which is assumed to lead to reporting of more income [46,47]. Information on legitimate power on the other hand includes the perception of the tax authority as doing a good job and therefore may affect tax morale and social norms [48]. Especially the source of information [33] may be relevant for the perception of social norms. Information on tax authority’s power through informal communication with other taxpayers may affect the perception of social norms of cooperation, which are relevant determinants of tax compliance [49]. Further research needs to clarify if the impact of additional information on tax evasion is mediated through the perception of social norms or tax morale. The current studies cannot explain whether additional information on power outweighs economic determinants or rather shapes the interpretation of economic key figures. It is needed to be investigated further, whether taxpayer still believe in objective determinants, although they receive additional information.

It might be argued that the descriptions of the tax authorities in experiments 2–4 induce a demand effect that participants feel forced to act according to the descriptions (c.f., [50,51,52]). Although in principle this is correct, it is also the actual objective of the experiments to investigate this effect (c.f., [53]). Further, the experimental setting reproduces taxpayers’ reality, who are confronted rather with descriptions and stories of tax authorities than with economic key figures. Thus, the experimental manipulation is increasing external validity (c.f., [54]).

In reality, taxpayers are confronted with a lot of information displaying the tax authority of their country through several information sources. The manipulation in the current studies compromises information on tax authority’s coercive and legitimate power, but cannot fully represent the complexity of the presentation of the authority in reality. Field experiments as well as further laboratory experiments should focus on how taxpayers deal with the wealth of information they receive through various sources and how tax authority’s power should be displayed in order to be recognized by taxpayers.

Taxpayers’ beliefs about tax authority’s power may not only be modified by information about the authority, but are likely to change according to the experience with the tax authority. Beliefs may especially change after taxpayers experienced an audit. In the current study, participants only got to know at the end, which period was selected for tax audits and if they are audited and fined or not. To further eliminate the impact of real life beliefs on the perception of the fictitious tax authority, students were used as experimental participants, like in similar tax experiments [31], as they are naïve regarding tax payments. The real life beliefs might have interfered with the manipulated perception of the fictitious tax authority. It can be assumed that actual information about the tax authority, for instance, through information letters or newspaper articles, might be even more effective in collecting taxes than a simple description in an experimental setting, and assure robustness and generalization of the findings. As taxpayers usually lack information about the exact audit probabilities [55], official information of the tax authority as well as unofficial information from other taxpayers may be an even more important information source for tax decisions [33]. This certainly needs to be investigated further.

Concluding, the insight gained from the current study has a considerable scientific as well as practical impact. It clearly demonstrated that despite the provision of objective economic figures, such as audit probability and fines, the subjective perception of power of the tax authority impacts tax payments. From a scientific perspective, this finding is particularly important for field experiments or laboratory experiments, where descriptions or vignettes are used to inform participants about the tax authority. According to the current results, researchers should pay attention on how they supply information since more information is needed to fully understand the interaction of different qualities of power. From a practical perspective, the study sheds some light on the effect of the perception of the tax authority’s power on tax compliance. Perception of power is modified by the information provided by the tax authority. Thus, sending letters or changing the presentation by means of public information sources are a field for future research [1]. As audits are costly for the tax authority and the government and large fines are recommended to be utilized only when absolutely necessary [56], such strategies could be considered as alternatives to lower the cost for levying taxes [4,5,26].

## Supporting Information

S1 Data Raw data for Experiment 1. (SAV)

S2 Data Raw data for Experiment 2 and 3. (SAV)

S3 Data Raw data for Experiment 4. (SAV)

S1 Table Items for participants`beliefs about tax authority`s power. (PDF)
