# Supplementary material for: Does the Sole Description of a Tax Authority Affect Tax Evasion? - The Impact of Described Coercive and Legitimate Power
Source: PLoS One. 2015 Apr 29;10(4):e0123355. doi: 10.1371/journal.pone.0123355 (PMC4414547; doi:10.1371/journal.pone.0123355)
Supplement: S1 Table — (PDF) [file pone.0123355.s004.pdf]

Items for participants` beliefs about tax authority's power:

Items are translated from the original German into English.

English	German
Coercive power	
I believe that the tax authority …	*Ich glaube, dass die Steuerbehörde …*
severely punishes tax evaders.	SteuerhinterzieherInnen streng bestraft.
enforces its demands through audits and penalties.	ihre Forderungen durch Kontrollen und Strafen durchsetzt.
punishes severely, when it finds a mistake.	hart bestraft, wenn sie einen Fehler findet.
persecutes taxpayers with audits and fines.	SteuerzahlerInnen mit Kontrollen und Strafen verfolgt.

English	German
Legitimate power	
I believe that the tax authority …	*Ich glaube, dass die Steuerbehörde …*
depends on taxpayers who fill out their tax forms correctly in order to work efficiently.	davon abhängig ist, dass die SteuerzahlerInnen ihre Steuerformulare sorgfältig ausfüllen, um effizient arbeiten zu können.
knows, how to give good advice to taxpayers.	weiß, wie man SteuerzahlerInnen gut berät.
provides taxpayers with professional advice.	für eine professionelle Beratung der SteuerzahlerInnen sorgt.
should receive a diligently completed tax return if past tax returns were often carelessly or erroneously completed.	eine gewissenhaft ausgefüllte Steuererklärung bekommen sollte, wenn in der Vergangenheit die Steuererklärung oft fehlerhaft ausgefüllt wurde.
arranges comprehensible procedures for the collection of taxes.	für nachvollziehbare Abläufe bei der Einhebung von Steuern sorgt.
is an institution taxpayers feel obliged to cooperate	eine Institution ist, bei der sich die SteuerzahlerInnen

with because it has rendered them many services in the past, e.g., child support.	zur Kooperation verpflichtet fühlen, weil sie ihnen in der Vergangenheit schon viele Transferleistungen, z.B.: Kinderbeihilfe etc., ausgezahlt hat.
holds counseling sessions, at which the correct way to file tax returns is demonstrated in minute detail so that taxpayers can learn.	in Beratungsgesprächen genau darstellt, wie die Steuererklärungen ausgefüllt werden müssen, damit die SteuerzahlerInnen daraus lernen können.
is an institution that taxpayers feel obliged to because it has accommodated them in past audits.	eine Institution ist, der sich die SteuerzahlerInnen verpflichtet fühlen, weil sie ihnen in der Vergangenheit bei Kontrollen entgegengekommen ist.
ensures that concerns of taxpayers are processed efficiently and fast.	dafür sorgt, dass die Belange von SteuerzahlerInnen effizient und rasch bearbeitet werden.
can only provide good advice and information if taxpayers are honest.	nur durch die Ehrlichkeit der SteuerzahlerInnen gut beraten und informieren kann.
deserves an honest tax return if tax obligations have often not been met in the past.	eine ehrliche Steuererklärung verdient hat, wenn in der Vergangenheit sehr oft der Steuerpflicht nicht nachgekommen wurde.
has the right to persecute tax evasion.	das Recht hat, Steuerhinterziehung zu verfolgen.
should receive correct tax returns if previous tax returns were completed very carelessly.	eine korrekte Steuererklärung bekommen sollte, wenn in der Vergangenheit die Steuererklärung sehr schlampig ausgefüllt wurde.
needs taxpayers’ cooperation to fulfill its duties properly.	die Kooperation der SteuerzahlerInnen benötigt, um ihre Aufgaben ordnungsgemäß ausführen zu können.
is appreciated by taxpayers for providing comprehensive information and advice.	von den SteuerzahlerInnen dafür gewürdigt wird, dass sie umfangreich informiert und berät.
has the legal obligation to collect taxes.	die rechtliche Pflicht hat, Steuern einzuheben.
is valued by taxpayers for the high standards it sets itself regarding the collection of taxes.	von den SteuerzahlerInnen dafür geschätzt wird, dass sie sich für die Steuereinhebung hohe Standards setzt.
is valued by taxpayers for using audits additionally to advise taxpayers.	von den SteuerzahlerInnen dafür geschätzt wird, dass sie Kontrollen auch zu Beratungszwecken einsetzt.
is legally obligated to advise taxpayers on tax-related matters.	rechtlich verpflichtet ist, SteuerzahlerInnen bei Steuerangelegenheiten zu beraten.

supplies comprehensive information that helps taxpayers orient themselves as to how to pay their taxes correctly.	ein übersichtliches Informationsangebot hat, das SteuerzahlerInnen hilft, sich zurechtzufinden, wie sie ihre Steuern richtig abführen.
is an institution taxpayers feel obliged to because it has considered their concerns in the past.	eine Institution ist, der sich die SteuerzahlerInnen verpflichtet fühlen, weil sie ihre Anliegen in der Vergangenheit berücksichtigt hat.
occupies employees, who inform comprehensively about tax issues.	MitarbeiterInnen hat, die über Steuerangelegenheiten umfangreich informieren.
